# Policy to expand hospital utilization in disadvantaged areas in Indonesia: who should be the target?

**DOI:** 10.1186/s12889-022-14656-x

**Published:** 2023-01-03

**Authors:** Agung Dwi Laksono, Hario Megatsari, Felly Philipus Senewe, Leny Latifah, Hadi Ashar

**Affiliations:** 1 National Research and Innovation Agency, Republic of Indonesia, Jakarta, Indonesia; 2 Faculty of Public Health, Universitas Airlangga, Surabaya, Indonesia

**Keywords:** Disadvantaged areas, Hospital utilization, Healthcare evaluation, Healthcare access, Public health

## Abstract

**Background:**

The disadvantaged areas are one of the government’s focuses in accelerating development in Indonesia, including the health sector. The study aims to determine the target for expanding hospital utilization in disadvantaged areas in Indonesia.

**Methods:**

The study employed the 2018 Indonesian Basic Health Survey data. This cross-sectional study analyzed 42,644 respondents. The study used nine independent variables: residence, age, gender, marital, education, employment, wealth, insurance, and travel time, in addition to hospital utilization, as a dependent variable. The study employed binary logistic regression to evaluate the data.

**Results:**

The results found that average hospital utilization in disadvantaged areas in Indonesia in 2018 was 3.7%. Urban areas are 1.045 times more likely than rural areas to utilize the hospital (95% CI 1.032–1.058). The study also found age has a relationship with hospital utilization. Females are 1.656 times more likely than males to use the hospital (95% CI 1.639–1.673). Moreover, the study found marital status has a relationship with hospital utilization. The higher the education level, the higher the hospital utilization. Employed individuals have a 0.748 possibility to use the hospital compared with those unemployed (95% CI 0.740–0.757). Wealthy individuals have more chances of using the hospital than poor individuals. Individuals with all insurance types are more likely to utilize the hospital than those uninsured. Individuals with travel times of ≤ 1 h are 2.510 more likely to use the hospital than those with > 1 h (95% CI 2.483–2.537).

**Conclusion:**

The specific targets to accelerate the increase in hospital utilization in disadvantaged areas in Indonesia are living in a rural area, being male, never in a union, having no education, being employed, being the poorest, uninsured, and having a travel time of > 1 h. The government should make a policy addressing the problem based on the research findings.

## Background

Hospital is an advanced-level referral health facility (ALRHF) in the National Health Insurance (NHI) program that handles individual health problems and consists of specialist-level health services, subspecialty; advanced outpatient care; and inpatient unique treatment rooms [1, 2]. Furthermore, as an ALRHF, hospital services include administrative services, services for drugs and medical consumables, and advanced diagnostic support services according to medical indications. Moreover, the services include medical rehabilitation services, blood services, clinical forensic medicine services, and services for the bodies of patients who died in health facilities [2].

Upon referral from a first-level health facility (FLHF), citizens who become a member of the NHI program can use the hospital as an ALRHF, except for emergencies, disasters, geographical considerations, consideration of the availability of facilities, and the specificity of the patient’s health problems [2]. Health facilities included in the FLHF are Health Center Institution (*Puskesmas*); general practitioner and dentist; private and government-owned primary clinics; as well as class D hospitals in collaboration with Social Security Administering Agency Health [2]. Based on the regulations of the Indonesian Health Ministry, *Puskesmas* is a health service facility that organizes public and first-level individual health efforts by prioritizing promotive and preventive actions in their working areas [3]. To help all Indonesians access the health services needed at affordable costs and realize universal health coverage (UHC), we expect the NHI program to have a referral system [4, 5].

Since Indonesia is an archipelagic country, it has an impact on efforts to equally distribute health services; therefore, the challenges to implementing UHC are not only regarding costs but also as regards the geographical aspect [6]. Moreover, there are disadvantaged areas and districts where sites and communities are less developed than other regions on a national scale [7]. The government determines the status of underdeveloped areas in a district concerning the economic aspects of the community, human resources, facilities and infrastructure, regional financial capacity, accessibility, and regional characteristics [7]. Based on the Indonesian Health Social Security Agency report, the coverage of UHC in 2018 was 208,149,019 individuals [8].

Based on the Presidential Regulation Number 63 of 2020, some condition categorized as disadvantaged areas is accessibility to vital infrastructure, including education and health. Regarding health facilities, accessibility in underprivileged areas remains unequal and has not been used optimally, and the number of doctors is minimal. Research conducted in Maurole, East Nusa Tenggara Province found that the availability of health services remained highly minimal. Some villages did not have the closest health facilities, such as the village health hut. Therefore, they had to go to a neighboring town or to the Maurole District Health Center, and almost all villages in Maurole had no physicians [9]. Several authors studied disadvantaged areas in the Bengkulu, South Sulawesi, and East Nusa Tenggara Provinces. The results revealed that the *Puskesmas* and hospital utilization remained not optimal for several reasons. Approximately 31% of the respondents admitted having a barrier to accessing health facilities. Approximately 14% claimed to be dissatisfied with their services at the Puskesmas, particularly due to the short operating hours of the *Puskesmas*. Moreover, approximately 47% claimed not receiving medical services [10].

Hospital utilization remains not optimal. Approximately 81% of the respondents reported that they knew which hospital to visit; however, 23.7% of the respondents admitted that receiving treatment at the hospital was difficult [10]. Besides the limited number of doctors, other issues faced by individuals in the East Nusa Tenggara area were the inadequate quality of health workers and limited health care [11]. One of the solutions to address these issues is advocating for the policy maker (government) to issue a policy that targets some problems based on the research findings (evidence-based approach).

Increasing access to health facilities in disadvantaged areas needs to be supported by the government as a policy maker. Therefore, we can realize that equitable access to health facilities in poor neighborhoods and achieve the Sustainable Development Goal commitment regarding UHC and particularly the “leave no one behind” commitment in terms of health. The “leave no one behind” approach in the health sector targets individuals who experience various obstacles to accessing health services and allow “health equity” to be implemented and fulfill the right to health [12]. To bring the “leave no one behind” approach into reality, the government needs to urgently prepare some aspects. These include data to identify individuals who are left behind, make individuals who are left behind and at risk of being left behind as a priority to understand what they need, and focus policies on disadvantaged communities and what can improve their welfare [13]. To realize the “leave no one behind” commitment in the health sector, the limitation of community access and use of FLHF and ALRHF as well as the minimum number of doctors in disadvantaged areas are the priorities that the government needs to pay attention to [9–11]. This study aimed to determine the target for expanding hospital utilization in disadvantaged areas in Indonesia.

## Materials and methods

### Data source

This study analyzed the 2018 Indonesian Basic Health Survey data. The survey was a national-scale cross-sectional survey run by the Republic of Indonesia’s Ministry of Health. The 2018 Indonesian Basic Health Survey collected data from May to July 2018. Furthermore, the survey used household and individual instruments. Nationally, the household and individual response rates were 95.58% and 93.20%, respectively.

The survey consisted of individuals from Indonesian households. The survey took the sample structure from the 2018 National Socio-Economic Survey performed in March 2018. Furthermore, the survey visited a target sample of 300,000 homes from 30,000 census blocks in the 2018 Socio-Economic Survey (run by the Central Statistics Agency) [14].

The 2018 Indonesian Basic Health Survey used the probability proportional to size (PPS) method, employing systematic linear sampling in the following two stages:

1. Stage 1: Implicit stratification based on welfare strata of all census blocks resulting from the 2010 Population Census. The PPS method chose the sample survey as the sampling frame for selecting census blocks from a master frame of 720,000 census blocks from the 2010 Population Census, of which 180,000 were chosen (25%). The survey used the PPS method to determine numerous census blocks in each urban/rural strata per regency/city to create a Census Block Sample List. The survey chose a total of 30,000 Census Blocks.
2. Stage 2: using systematic sampling, select ten homes in each Census Block were selected with the highest implicit stratification of education completed by the Head of Household to preserve the representation of the diversity value of household characteristics. The survey asked all household members in the selected household as part of the 2018 Indonesian Basic Health Survey [14].

The study comprised a population of adults (≥ 15 years old) in disadvantaged areas in Indonesia. It described 42,644 respondents as a weighted sample based on the sampling methods.

### Setting

The study analyzes hospital utilization in underdeveloped areas in Indonesia. The government set out boundaries of disadvantaged areas in Presidential Regulation Number 63 of 2020 concerning the Determination of Underdeveloped Regions for 2020–2024. Based on the regulation, underdeveloped areas include 62 regencies in the following 11 provinces in Indonesia: North Sumatera Province (Nias, South Nias, North Nias, and West Nias), West Sumatera Province (Mentawai Islands), South Sumatera Province (North Musi Rawas), Lampung Province (West Pesisir), West Nusa Tenggara Province (North Lombok), East Nusa Tenggara Province (West Sumba, East Sumba, Kupang, East Timor Tengah, Belu, Alor, Lembata, Rote Ndao, Central Sumba, Southwest Sumba, East Manggarai, Sabu Raijua, and Malaka), Central Sulawesi Province (Donggala, Tojo Una-una, and Sigi), Maluku Province (West Maluku Tenggara, Aru Islands, West Seram, East Seram, Southwest Maluku, and South Buru), North Maluku Province (Sula Islands, and Taliabu Island), West Papua Province (Wondama Gulf, Bintuni Gulf, South Sorong, Sorong, Tambrauw, Maybrat, South Manokwari, and Arfak Mountains), and Papua Province (Jayawijaya, Nabire, Paniai, Puncak Jaya, Boven Digoel, Mappi, Asmat, Yahukimo, Bintang Mountains, Tolikara, Keerom, Waropen, Supiori, Great Mamberamo, Nduga, Lanny Jaya, Central Mamberamo, Yalimo, Puncak, Dogiyai, Intan Jaya, and Deiyai).

### Dependent variable

The dependent variable was hospital utilization, which was defined as an adult’s access to outpatient or inpatient hospitals. Hospital utilization consists of the following two classes: unutilized and utilized (outpatient and inpatient). Conversly, outpatient hospitalizations were limited to the previous month, whereas limited to inpatient hospitalizations were limited to the last year. The survey asked respondents to correctly recall outpatient and inpatient episodes in the survey [14].

### Independent variables

The study employed nine independent variables, including the type of residence, age, gender, marital status, education level, employment status, wealth status, health insurance ownership, and travel time to the hospital. The type of residence consists of urban and rural areas. Moreover, the study used the Indonesian Central Statistics Agency’s provisions for urban rural categorization.

The study calculated age using the respondent’s last birthday, and male and female were the two genders. Additionally, based on their marital status, the survey divided the respondents into the following three categories: never in union, married/living with a partner, and divorced/widowed.

The acknowledgment of the respondent’s most recent diploma determined their education status. The survey covered the following four formal education levels: didn’t get formal education, primary, secondary, and higher. Meanwhile, the employment status consisted of unemployed and employed.

The survey used the wealth index formula to identify the wealth status in the study. It calculated the wealth index using a weighted average of a family’s total spending. Meanwhile, the survey computed the wealth index using primary household expenditures, including health insurance, food, and lodging. Furthermore, the survey divided the income index into the following five categories: poorest, poorer, middle, richer, and richest [15, 16].

Moreover, the survey categorized health insurance ownership into the following four categories: uninsured, government-run insurance, private-run insurance, and having both insurances (government-run and private-run insurance). Moreover, the travel time to the hospital consisted of ≤ 1 and > 1 h.

### Data analysis

The Chi-square test was used in the early phases of the sample to create a bivariate comparison for the dichotomous variable. Simultaneously, a T-test was used for the continuous variable (age). Additionally, a collinearity test was used to determine the relationship between the independent variables in the final regression model. The analysis used a binary logistic regression in the study’s last point. The previous test was used in the study to examine the multivariate relationship between all independent factors and hospital utilization. All statistical analyses were performed using IBM SPSS 26.

## Results

**Fig. 1:**
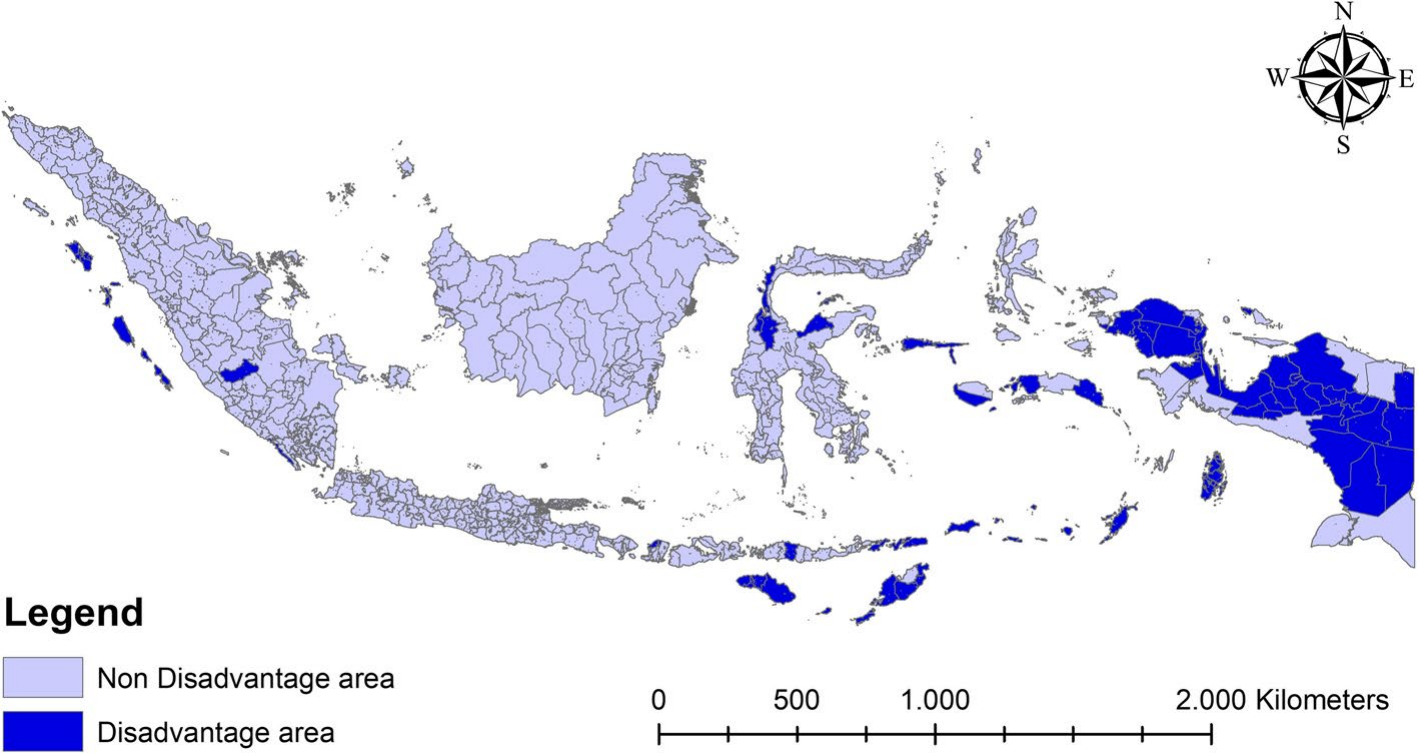
. Distribution of Hospital Utilization in Indonesia.

The results revealed that the average hospital utilization in disadvantaged areas in Indonesia in 2018 was 3.7% (See Fig. 1). The descriptive statistics of the respondents are shown in Table 1. Individuals who did not utilize hospitals were mainly from urban and rural areas. Meanwhile, those who lived in rural areas had a slightly older average age than those in urban areas. Moreover, regarding gender, there were more females than males in both urban and rural areas.

**Table 1:** Descriptive statistics of respondents (*n* = 42,644)

Older adult Characteristics	Hospital utilization		*p*-value
	**Unutilized (***n*** = 41,053)**	**Utilized** **(***n*** = 1,591)**	
**Type of residence**			< 0.001
Urban	11.8%	20.9%	
Rural	88.2%	79.1%	
**Age (mean)**	(37.15)	(42.42)	< 0.001
**Gender**			
Male	50.9%	38.0%	
Female	49.1%	62.0%	
**Marital status**			< 0.001
Never in union	22.7%	11.4%	
Married/living with a partner	70.2%	78.7%	
Divorced/widowed	7.0%	9.9%	
**Education level**			< 0.001
No formal education	16.0%	10.4%	
Primary	58.4%	53.2%	
Secondary	19.2%	24.8%	
Higher	6.4%	11.7%	
**Employment status**			< 0.001
Unemployed work	29.8%	34.1%	
Employed	70.2%	65.9%	
**Wealth status**			< 0.001
Poorest	34.9%	23.4%	
Poorer	20.7%	17.2%	
Middle	16.8%	21.2%	
Richer	14.0%	16.8%	
Richest	13.6%	21.4%	
**Health insurance**			< 0.001
Uninsured	28.5%	17.3%	
Government-run insurance	70.8%	81.6%	
Private-run insurance	0.5%	0.7%	
Government-run and private-run insurance	0.2%	0.3%	
**Travel time**			< 0.001
≤ 1 h	41.4%	68.0%	
> 1 h	58.6%	32.0%	

Individuals who lived in rural areas led in both categories of hospital utilization in disadvantaged regions in Indonesia, as shown in Table 1. Regarding age, those who utilized the hospital had an average age older than those who unutilized the hospital. Furthermore, males led in the unutilized hospital group, whereas females in the utilized hospital group.

Regarding marital status, married or living with a partner dominated both categories of hospital utilization. Conversely, primary education ruled the unutilized and utilized the hospital groups. Moreover, employment led to both types of hospital utilization.

Regarding wealth status, the poorest occupied both groups of hospital utilization. Conversely, government-run insurance dominated all hospital utilization categories based on health insurance ownership. Meanwhile, those with a travel time of > 1 h led in the unutilized hospital category, whereas those with a travel time of ≤ 1 h ruled in the utilized hospital category.

Subsequently, a collinearity test was performed. Based on the test results, no significant association between the independent variables was noted. All variables had a tolerance value of > 0.10; however, the variance inflation factor value for all factors was < 10.00. According to the data, the regression model revealed no signs of multicollinearity.

The binary logistic regression of hospital utilization in disadvantaged areas in Indonesia is presented in Table 2. This study used “hospital unutilized” as a reference in this final stage.

**Table 2:** The result of binary logistic regression of hospital utilization in disadvantaged areas in Indonesia in 2018 (*n* = 42,644)

PREDICTORS	*p*-value	Hospital utilization		
		**AOR**	**95% CI**	
			**Lower bound**	**Upper bound**
Residence: Urban	< 0.001	1.045	1.032	1.058
Residence: Rural	-	-	-	-
Age	< 0.001	1.022	1.021	1.022
Gender: Male	-	-	-	-
Gender: Female	< 0.001	1.656	1.639	1.673
Marital status: Never in union	< 0.001	0.636	0.621	0.652
Marital status: Married/living with partner	< 0.001	1.080	1.062	1.099
Marital status: Divorced/widowed	-	-	-	-
Education: No formal education	-	-	-	-
Education: Primary	< 0.001	1.589	1.564	1.615
Education: Secondary	< 0.001	2.207	2.167	2.248
Education: Higher	< 0.001	2.459	2.406	2.513
Employment: Unemployed	-	-	-	-
Employment: Employed	< 0.001	0.748	0.740	0.757
Wealth: Poorest	-	-	-	-
Wealth: Poorer	< 0.001	1.079	1.063	1.095
Wealth: Middle	< 0.001	1.538	1.517	1.560
Wealth: Richer	< 0.001	1.323	1.303	1.344
Wealth: Richest	< 0.001	1.572	1.549	1.596
Insurance: Uninsured	-	-	-	-
Insurance: Government-run	< 0.001	1.802	1.780	1.825
Insurance: Private-run	< 0.001	1.507	1.423	1.595
Insurance: Government-run and private-run	< 0.001	2.137	1.963	2.326
Travel time: ≤ 1 h	< 0.001	2.510	2.483	2.537
Travel time: > 1 h	-	-	-	-

*Abbreviations: AOR *adjusted odds ratio, *CI *confidence interval, *LB *lower bound, *LB *lower bound

Table 2 shows that those who lived in urban areas are 1.045 times more likely to utilize the hospital than those who lived in rural areas (95% confidence interval [CI]: 1.032–1.058). Furthermore, the results demonstrated that age was significantly correlated with hospital utilization in the disadvantaged regions of Indonesia.

Regarding gender, females were 1.656 more likely to use the hospital than males (95% CI: 1.639–1.673). Meanwhile, regarding marital status, those who were never in union were 0.636 times less likely to utilize the hospital than those who were divorced/widowed (95% CI: 0.621–0.652). Moreover, individuals who were married or living with a partner were 1.080 times more likely to use the hospital than those who were divorced/widowed (95% CI: 1.062–1.099).

Table 2 shows that regarding the education level, the higher the level of education, the higher the hospital utilization in disadvantaged areas in Indonesia. Furthermore, those who were employed were 0.748 times less likely to use the hospital than those who were unemployed (95% CI: 0.740–0.757). Additionally, all individuals with a wealthy status had more likelihood of using the hospital than those with the poorest status in disadvantaged areas in Indonesia.

Regarding health insurance ownership, in disadvantaged areas in Indonesia, individuals with all types of insurance were more likely to utilize the hospital than those who were uninsured. Moreover, those with ≤ 1 h travel time to the hospital were 2.510 times more likely to utilize the hospital than those with > 1 h travel time (95% CI: 2.483–2.537).

## Discussion

This study aimed to determine hospital utilization factors for disadvantaged areas in Indonesia. We anticipate that understanding vulnerable characteristics in underdeveloped regions can help the government accelerate access and distribution of health services in those areas. In addition to assigning a presidential regulation to disadvantaged regions (previously remote, borders, and islands regions), the government issued several affirmative health policies to narrow the gap with the most disadvantageous areas.

*Nusantara Sehat* (NS), or Healthy Archipelago, is an integrated health effort covering prevention, promotion, and curative aspects through special assignments. A team-based health worker improved access to health services in disadvantaged, border, and island regions, enhancing the public health index [17, 18]. Moreover, the government in remote areas also implemented several health services, including flying doctor services, walking doctors, floating health center, mobile clinic, *Bhaskara Jaya Operation* [19], hospital aid vessels [20], and flying ambulances [21]. The problem still exists in the distribution and continuity of service in disadvantaged areas [18, 21].

The results have shown that urban areas have more probabilities to utilize the hospital than rural areas in disadvantaged regions in Indonesia. This is consistent with the results of a study conducted in France and China, which showed that access to services is higher for people in large urban hospitals [22, 23]. The disparities in rural areas’ hospital utilization could reflect the lack of equal opportunities and facilities due to the more limited facilities, health policies, and the development of healthcare systems in a region [23, 24].

The travel time indicator also proves the geographical factors related to hospital utilization in this research. Those with ≤ 1 h travel time have more possibilities to utilize the hospital than those with > 1 h in disadvantaged areas in Indonesia. This is similar to a study in the Netherlands that showed travel time as the most crucial determinant in hospital selection [25]. Travel time is related to distance and mobility. A study conducted in Florida and Beijing showed that the distance to the nearest hospital affects the utilization of hospital services; however, the effect is small [26–28]. A study conducted in India found that more comprehensive and reasonable care is found in facilities with a higher level of health care, such as in-district or provincial hospitals, in contrast to lower-level healthcare facilities in the regions, which affects patients’ mobility [29]. Other studies conducted in disadvantaged areas in Indonesia also revealed significant differences in access to maternal and child health services between remote and very remote areas [18]. The travel time impacted the efficiency of health services, including immediate care accessibility and affordability. This condition significantly influenced a more vulnerable population, including the poorest and the more remote. The government needs to rationally and equally allocate the existing resources, facilities, and infrastructure, including human medical resources, between areas [30].

Besides geographic factors, the difference in hospital utilization also came from the demand side, including socioeconomic and demographic characteristics [31, 32]. This study found that individuals with all types of insurance are more likely to utilize the hospital than those who are uninsured in disadvantaged areas in Indonesia. A previous study found that healthcare services, including hospital utilization, were higher for patients with insurance and even higher for those with comprehensive health insurance [33]. The critical objective of the health insurance scheme is to empower the community based on cooperation [34]. Indonesia’s UHC scheme is the largest single-payer scheme globally, covering 203 million individuals, thereby improving coverage, use, and delivery efficiency of health services [35, 36].

Meanwhile, individuals with a wealthy status have more chances to use the hospital than the poorest in disadvantaged areas in Indonesia. A study conducted in Mexico showed more medical visits and inpatient utilization in the middle and upper classes [34]. Individuals from the middle and upper classes use more health services for chronic disease treatment that require more intensive care. Furthermore, the study found that living standards, health insurance, and education mediate the wealth status on health care services utilization. Additionally, a study conducted in California demonstrated that high-income individuals used hospitals more than those with low income [37]. However, when age and occupation factors were included, a slight reduction effect was observed [38].

Regarding occupation, a previous study found a higher hospital utilization among the unemployed population in disadvantaged areas. This is similar to a study conducted in the United States from 2007 to 2009, which showed higher unemployment increased healthcare utilization spending among Medicaid beneficiaries. Therefore, higher unemployment healthcare spending was related to insurance coverage [39]. Regardless of UHC’s success in Indonesia, which has improved health equity and access, there was a missing middle group in wealth quintiles Q2–Q3. The lower-middle-income group has the highest number of uninsured individuals, and the employed could be in that quintile. Moreover, a previous study found that unemployment is related to lower health status, which could increase the probability of hospital care [40].

This study found a higher hospital utilization in females and the married population. A previous study in an underdeveloped area in India revealed that hospital utilization in females and the married population is closely related to ante- and perinatal care utilization [41]. Several countries and regions, including Indonesia [42, 43], sub-Saharan Africa [44], and India [41], showed increased participation of females in ante- and perinatal care. In the context of health service equality, there remains a demographic disparity in the disadvantaged area regarding maternal care utility coverage in Indonesia. In 2011, to lower the prevalence of maternal death, the Indonesian government issued universal coverage for childbirth insurance called Jampersal. It was an expansion of Public Health Insurance (Jamkesmas); however, the beneficiaries were not only the poor population. The Jampersal recipients include pregnant females, mothers in labor, and postpartum females without other health insurance coverage (from immediate post-delivery to 42 days after delivery); the financing also covers neonates (0–28 days old) [38, 45]. A deeper analysis of the underprivileged in India showed that hospital care utilization differed between females and males. Females access hospital care less than males in neutral-gender health care [41, 46]. The lower rate of male hospital utilization could also reflect the gap between female-related and gender-neutral healthcare hospital utilization.

The higher hospital utilization among the married population could also be attributable to a more effective pattern of hospital utilization, as suggested by a previous study [47]. The married population received higher social support, lowering the risk of psychiatric problems [48]. A previous study showed unmarried individuals as a more vulnerable population in terms of health care behavior (e.g., higher engagement in risky health behavior), health care access (reduced health resources such as disposable income and health insurance), and health status [49, 50]. Unmarried individuals need more assistance to acquire better health resources, make better decisions on healthy behavior, and have unique health care benefits.

This study found that age is also related to hospital utilization. This result is consistent with that of a study conducted in the Netherlands and China, which revealed that age is the most critical factor influencing medical service behavior [25]. Higher age was related to more experience and maturity level of the individual in decisions making. Adults use more health services than younger individuals [16].

Moreover, the higher education level is related to higher hospital utilization in disadvantaged areas in Indonesia. The health literacy factor could explain the relationship between education and hospital utilization. The previous study found that health literacy is related to higher hospital utilization [51], and it mediates educational attainment with the higher benefits from the healthcare setting [52, 53]. Individuals with higher healthcare literacies could have more resources (knowledge, communication, and relation) and be more autonomous in choosing healthcare facilities and treatment [54]. This finding is consistent with those of previous studies conducted in several European countries [53, 55] and other research conducted Indonesia [42].

Based on the result of this study, some potential implications for public health policies are noted. Some policy implications are that the government should pay attention to some aspects related to the disadvantaged areas, including education, infrastructure, and ownership of health insurance. Furthermore, the government also should take actions to overcome the abovementioned aspects; for example, regarding the aspect of education aspect, the government may launch a program that accelerates the education level for the community.

### Strength and limitation

The study examines a large amount of data to represent information based on a disadvantaged area basis. Conversly, it examines secondary data; therefore, this study limited analyzed variables to acceptable ones. Other factors related to hospital consumption discovered in previous studies, such as the cost of services, travel cost to the hospital, and the kind of disease, cannot be investigated [42, 56, 57].

## Conclusion

This study revealed eight factors related to hospital utilization in disadvantaged areas in Indonesia, that is, the type of residence, gender, marital status, education level, employment status, wealth status, health insurance ownership, and travel time to the hospital. Moreover, the specific targets to accelerate the increase in hospital utilization in disadvantaged areas in Indonesia are living in a rural area, being male, never in a union, having no education, being employed, being the poorest, uninsured, and having a travel time of > 1 h to the nearest hospital.

## Data Availability

The data that support the findings of this study are available from the Ministry of Health of the Republic of Indonesia but restrictions apply to the availability of these data, which were used under license for the current study, and so are not publicly available. Data are however available from the corresponding author upon reasonable request and with permission of the Ministry of Health of the Republic of Indonesia.

## Abbreviations

NHI: National Health Insurance
UHC: Universal Health Coverage
SDGs: Sustainable Development Goals
PPS: probability proportional to size
VIF: variance inflation factor
AOR: adjusted odds ratio
CI: confidence interval
LB: lower bound
UB: upper bound
